# Sex-Specific Correlations of Individual Heterozygosity, Parasite Load, and Scalation Asymmetry in a Sexually Dichromatic Lizard

**DOI:** 10.1371/journal.pone.0056720

**Published:** 2013-02-25

**Authors:** Pei-Jen L. Shaner, Ying-Ru Chen, Jhan-Wei Lin, Jason J. Kolbe, Si-Min Lin

**Affiliations:** 1 Department of Life Science, National Taiwan Normal University, Taipei, Taiwan; 2 Department of Biological Sciences, University of Rhode Island, Kingston, Rhode Island, United States of America; Université de Sherbrooke, Canada

## Abstract

Heterozygosity-fitness correlations (HFCs) provide insights into the genetic bases of individual fitness variation in natural populations. However, despite decades of study, the biological significance of HFCs is still under debate. In this study, we investigated HFCs in a large population of the sexually dimorphic lizard *Takydromus viridipunctatus* (Lacertidae). Because of the high prevalence of parasitism from trombiculid mites in this lizard, we expect individual fitness (i.e., survival) to decrease with increasing parasite load. Furthermore, because morphological asymmetry is likely to influence individuals' mobility (i.e., limb asymmetry) and male biting ability during copulation (i.e., head asymmetry) in this species, we also hypothesize that individual fitness should decrease with increasing morphological asymmetry. Although we did not formally test the relationship between morphological asymmetry and fitness in this lizard, we demonstrated that survival decreased with increasing parasite load using a capture-mark-recapture data set. We used a separate sample of 140 lizards to test the correlations between individual heterozygosity (i.e., standardized mean *d^2^* and HL based on 10 microsatellite loci) and the two fitness traits (i.e., parasite load and morphological asymmetry). We also evaluated and excluded the possibility that single-locus effects produced spurious HFCs. Our results suggest male-only, negative correlations between individual heterozygosity and parasite load and between individual heterozygosity and asymmetry, suggesting sex-specific, positive HFCs. Male *T. viridipunctatus* with higher heterozygosity tend to have lower parasite loads (i.e., higher survival) and lower asymmetry, providing a rare example of HFC in reptiles.

## Introduction

Individual genetic diversity is the degree of difference between alleles from homologous chromosomes in a diploid organism. The heterozygous advantage hypothesis originated from the expectation that heterozygous individuals have higher relative fitness than homozygous ones [1]. Although heterozygosity-fitness correlations (HFCs) have been studied for decades [2], inconsistent HFCs were reported across different populations and taxa, with weak overall effect sizes [3]–[6]. However, not all taxa are well represented in HFC studies, and intra-population patterns (e.g., different directions and strengths of HFC for different sex or age groups) have been relatively unexplored. Therefore, more empirical evidence, particularly from under-studied taxa and different demographic groups, is needed before the biological significance of HFC can be fully understood.

Reptiles as a group are under-represented in HFC studies. In one of the most comprehensive meta-analyses on HFC published to date [6], only one reptile species was represented among a total of 61 species. Given the few studies in reptiles, there is little evidence for HFC in this clade. For example, a study on the ornate dragon lizard (*Ctenophorus ornatus*) showed that offspring survival was not influenced by degree of inbreeding [7]. Another study on the pygmy bluetongue lizard (*Tiliqua adelaidensis*) did not find a correlation between heterozygosity and endoparasite load [8]. Similarly, several studies on tuatara (*Sphenodon punctatus*) failed to demonstrate correlations between heterozygosity and male territory size, reproductive success, or body size [9], [10]. The lack of HFC in previous studies may be explained by insufficient genetic or fitness variation in the study populations [11], [12], [13]. Therefore, a population with a large effective population size under strong selection may be more promising for HFC studies.

Parasites negatively impact host growth, nutritional status, and survival (e.g., [14]–[18]). Empirical evidence has demonstrated correlations between individual heterozygosity and parasite load [14], [17], [19], [20], showing that this trait is a suitable index of body condition in wild animals. However, not all studies agree with respect to the fitness reduction effects of parasites in lizard hosts (e.g., [21]). Therefore, we need to first establish the relationship between parasite load and host fitness before using it as a fitness trait.

On the other hand, lower genomic heterozygosity could produce individuals with higher morphological asymmetry, which could reduce fitness in reptiles [22]–[25]. For example, the physical performance of the Italian wall lizard (*Podarcis sicula*) was observed to decrease with increasing fluctuating asymmetry [25]. In addition, male quality and female mate preference had a negative relationship with male femoral pore asymmetry in the Iberian rock Lizard (*Lacerta monticola*) [22], [23]. Shine et al. [24] found that ventral scale asymmetry in the red-sided garter snake (*Thamnophis sirtalis parietalis*) reduced male reproductive success. Therefore, scalation asymmetry in reptiles might be used as another fitness trait in HFC studies.

**Figure 1:**
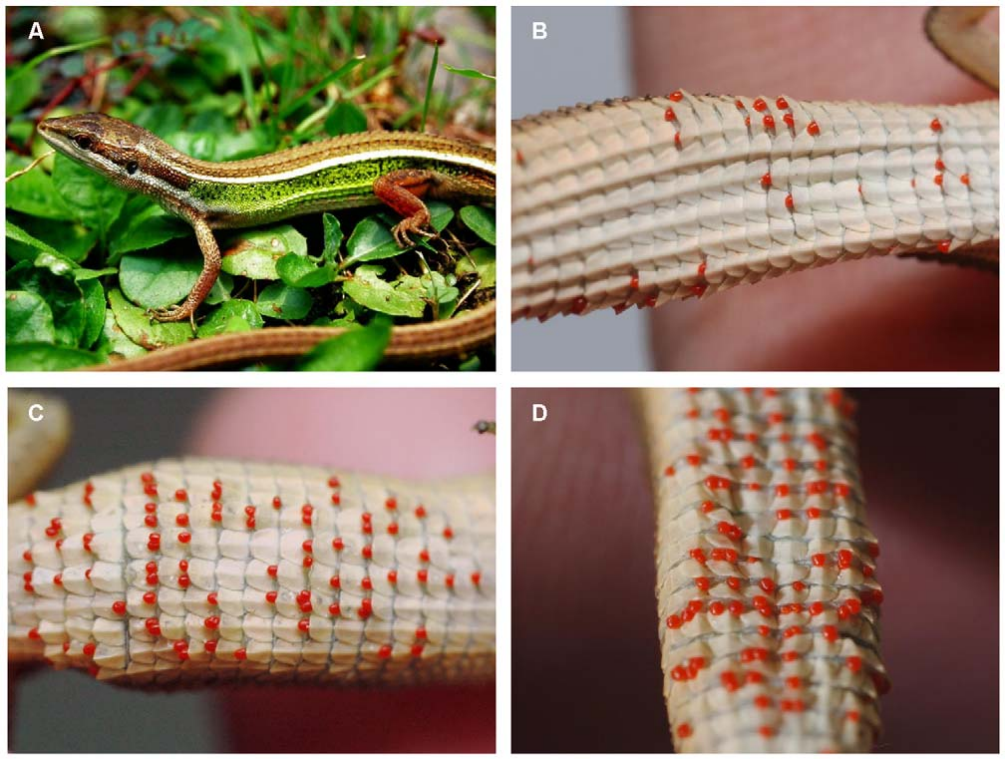
The *Takydromus* lizard and trombiculid mites. An adult male *Takydromus viridipunctatus* during the breeding season showing lateral green spots (A), and three different individuals with low (B), median (C) and high (D) infestation by trombiculid mites.

The goal of this study is to investigate HFC in a large population of green spotted grass lizard (*Takydromus viridipunctatus*). Males of this sexually dimorphic species have a significantly larger head size than do females [26], which might be advantageous in contests between males or to enable them to successfully grab a female during forced copulation [27]–[31]. In breeding seasons, males display shining green spots on the sides of the body, which sometimes cover the entire lateral surface (Figure 1A). During courtship, females spend more time close to those males with brighter coloration [32]. However, the key for successful copulation in this lizard might be the biting ability of the males, as reported in several other terrestrial lizards [27], [30], [31], [33]. Given its mating behaviors, we suspect that the symmetry of male head shape plays a crucial role in delivering a strong and effective copulatory bite. This species is specialized in the subtropical grassland habitats of early succession stage and is capable of forming extremely large and dense populations [26]. During a six-year (2006–2012) census of our current study population in Taiwan, several hundred individuals could be collected along a 500-meter transect during a single night, with more than 10,000 unique individuals marked over the six-year period (annual population size of 1,600 individuals; Lin, S.-M., unpublished data). In addition, parasitism from trombiculid mites (*Leptotrombidium* sp., Figure 1B–D) was monitored during these six years, and the results indicate a consistently high prevalence of parasitism (mean prevalence is 0.74, with an annual peak in July; Lin, S.-M., unpublished data).

Because of the high level of parasitism prevalence and forced copulation behaviors in this species, we expect to see strong correlations between individual heterozygosity and parasite load, and between individual heterozygosity and fluctuating asymmetry. Furthermore, given the distinct sexual dichromatism and potentially different reproductive costs between sexes in this lizard, we also expect to detect sex-specific HFC. Our specific predictions are: (1) parasite load should be negatively correlated with individual survival; (2) individual heterozygosity should be negatively correlated with parasite load and scalation asymmetry; and (3) these HFCs should be sex-specific.

## Materials and Methods

### Lizard Survey to Determine the Effects of Parasite Load on Lizard Survival

A monthly capture-mark-recapture (CMR) survey of *T. viridipunctatus* was conducted between July and November in 2008 and 2009 at Cape Jinshan on the northeastern coast of Taiwan (25°13′34″N, 121°38′55″E). The lizards were captured by hand at night along a 500-meter transect. All captured individuals were uniquely tagged by toe clipping, sexed, and aged, and the number of the trombiculid mites (*Leptotrombidium* sp.) on each lizard was counted before they were released back to the population. Only adult lizards (totally 1104 individuals) were included in the survival analysis to match the separate samples used in our analyses of heterozygosity-fitness correlations in adult lizards.

Two methods were conducted to evaluate the effect of parasites on survival of adult lizards during the breeding season. First, we used Spearman correlations to check the association between maximum capture interval (in month) and the mean parasite load (the mean number of mites from each capture of each lizard). We then used the Cormack-Jolly-Seber (CJS) model implemented in program MARK [34], [35] to estimate the survival of adult lizards as a linear function of mean number of trombiculid mites. The CJS model estimates two parameters: survival (φ) and recapture probability (p). Although φ is the probability that any given individual will have remained in the population and survived through a specific time period, and thus cannot be used to distinguish between mortality and emigration, our study site is surrounded by urban areas and ocean, making successful emigration unlikely. The time and sex dependency of survival and recapture probability were first evaluated with Akaike's Information Criterion, corrected for small sample sizes (AIC_c_), which suggested that survival is constant (φ.) or sex-varying (φ_sex_) and that recapture probability is time- and sex-varying (p_sex×t_; Table S1). We used these two models as base models (φ. p_sex×t_ and φ_sex_p_sex×t_) and constructed four additional models to test the effects of mites on the *T. viridipunctatus* survival functions: sex-varying intercepts and slopes of mean number of mites, sex-varying intercept and a constant slope of mean number of mites, a constant intercept and sex-varying slopes of mean number of mites, and constant intercept and slope of mean number of mites (Table 1). The fit of the more complex base model (φ_sex_p_sex×t_) was assessed with C? (≈χ^2^/df); a C? close to 1 indicates perfect model fit, and a C? smaller than 2 usually suggests a good fit. We used the three estimates of C? implemented in MARK to assess model fit: (1) C? from the program RELEASE, which provided an estimate of C? = 0.82 (RELEASE does not provide standard errors); (2) median C? = 1.04±0.02 (± SE); and (3) bootstrap C? = 1.57±0.02. All three estimates suggest a fairly good model fit. In addition, we visually assessed the distribution of the residuals using the residual deviance plot, which suggested that the residuals are randomly distributed around zero. Model goodness of fit cannot be directly assessed when individual covariates are incorporated in the CJS models. However, given that the base model had a good fit, we were able to test the effects of mite parasitism on lizard survival using likelihood ratio (LR) tests (i.e., determine whether the models with mean number of mites as a covariate significantly improved the model fit from the base models).

**Table 1:** Model selection from six candidate survival functions.

Model	AIC_c_	Model likelihood	Deviance	Number of parameters	LR test Chi.-sq.
*Models without individual covariates*					
1. logit(φ) = α	1661.6403	0.0000	1643.4727	9	NA
2. logit(φ*_m_*) = α*_m_*; logit(φ*_f_*) = α*_f_*	1661.3656	0.0000	1641.1606	10	NA
*Models with mean number of mites as an individual covariate*					
3. logit(φ) = α+β×(mean number of mites)	1624.9764	1.0000	1604.7714	10	38.701***
4. logit(φ*_m_*) = α_m_+β×(mean number of mites); logit(φ*_f_*) = α_f_+β×(mean number of mites)	1626.2046	0.5411	1603.9583	11	37.202***
5. logit(φ*_m_*) = α+β*_m_*×(mean number of mites); logit(φ*_f_*) = α+β*_f_*×(mean number of mites)	1627.0156	0.3607	1604.7693	11	38.703***
6. logit(φ*_m_*) = α*_m_*+β*_m_*×(mean number of mites); logit(φ*_f_*) = α*_f_*+β*_f_*×(mean number of mites)	1626.3365	0.5066	1602.0452	12	39.115***

The comparison of six survival functions with or without mean number of mites as an individual covariate for survival estimates of *Takydromus viridipunctatus*.

φ: survival; α: intercept; β: slope; m: males; f: females; ***: <0.0001. Survival functions were estimated in Cormack-Jolly-Seber models with time- and sex-varying recapture probabilities. The models with mean number of mites as an individual covariate (models 3–5) generally had lower AIC_c_ values and produced significantly better model fits than the reduced models (models 1&2), as evaluated by LR tests (model 1 versus 3, model 1 versus 5, model 2 versus 4, and model 2 versus 6).

### Lizard Sampling for Correlations among Individual Heterozygosity, Parasite Load, and Scalation Asymmetry

The toe-clipping method used in the long-term survey made it difficult to quantify scalation asymmetry (the scalation asymmetry scores of the toes are included as part of the asymmetry measure), one of the two fitness traits used in our HFC study. To obtain a sample of lizards with intact toes, we conducted a separate sample collection. To ensure that this sampling did not interfere with the survival estimates, we collected 140 adult lizards (78 males and 62 females) from a nearby location in July 2008. Although this sampling could potentially lower the lizard population size, the 2008–2009 survival estimates were based on lizards marked after this sampling was conducted. Compared to the estimated population sizes of 1,122 in 2008 and 1,066 in 2009 during these months (minimum number alive), a sample of 140 individuals was likely to represent only 12–13% of the population. In addition, we conducted the sampling approximately 100 meters from the survey transect to ensure that we removed only individuals at the boundary of the population for which we were estimating survival. We used only adult lizards without autotomized tails in this sampling to control for the potentially confounding effects of lizard body condition on mite infestation.

### Individual Heterozygosity

Genomic DNA was extracted from lizard muscle tissues using a modified LiCl method [36]. Ten microsatellite loci [37] were evaluated to estimate individual heterozygosity (Table S2). The polymerase chain reactions (PCRs) were set up in a volume of 10 µl containing 50–100 ng genomic DNA, 1× PCR buffer (PROMEGA), 0.15–0.2 µM each forward and reverse primers, 2.5 mM MgCl_2_, 0.2 mM dNTP, and 0.25 U Taq DNA polymerase (PROMEGA). PCR was carried out under standard conditions, with modifications for each locus [37]. The PCR products were electrophoresed in a MegaBASE™ 1000 autosequencer (Amersham Bioscience, New Jersey, USA) with size marker ET-400 (Amersham Bioscience New Jersey, USA). Alleles were scored manually in GENETIC PROFILER version 2.2 (Amersham Bioscience, New Jersey, USA). Tests for departure from Hardy-Weinberg equilibrium and linkage disequilibrium between pairs of loci were performed using Arlequin ver. 3.5 [38]. Two of the 10 microsatellite loci were found to be out of Hardy-Weinberg equilibrium. However, the analyses of the data with either eight or 10 microsatellite loci gave similar results. Therefore, we reported only the results based on all 10 microsatellite loci.

Individual heterozygosity was initially evaluated by three different approaches: standardized mean d-squared (standardized *d* ^2^), internal relatedness (IR), and heterozygosity weighted by locus (HL). Standardized mean *d* ^2^ is defined as squared distance divided by the maximum value observed on that locus, and then averaged across loci [39]. HL [40], an estimate of parental relatedness, is modified from the widely applied IR [41] but tends to outperform the latter. Because HL and IR are highly correlated (*r* ^2^ = 0.96) in this study, only HL was retained in the following analyses. Because standardized mean *d* ^2^ was considered less informative in recent studies [6], [13], we accept HFCs only when the results based on standardized mean *d^2^* and HL are congruent.

### Parasite Load

Parasite load, i.e., the number of parasites on a host, was measured by immediately counting the number of trombiculid mites attached to each individual lizard (Figure 1). These mites are relatively large in size (∼400 µm) and bright red in color. They attach themselves tightly to gaps between the ventral scales using their jaws and are therefore visible as a pattern of discrete spots. The morphological and behavioral characteristics of these mites allowed for an accurate count of parasite load. The parasite load was quantified using an identical protocol in the 2008–2009 survey and the separate sample of 140 lizards, and mean parasite load was calculated for individuals who were captured multiple times during the survey.

### Scalation Asymmetry

Scalation asymmetry was measured for the separate sample of 140 lizards by summing the absolute difference in scale numbers between the left and right sides across the chin shields, supralabials, infralabials, supraciliary scales, and supraocular scales in the head region and the subdigital lamellae of all fingers and toes of the limbs. The raw asymmetry scores ranged between 0 to 3 in the head region and 1 to 14 in the limbs. Therefore, we converted the limb asymmetry scores to a 0–3 scale using the following equation: (limb asymmetry score-1)/4.33. We added the head and normalized limb asymmetry scores to produce an overall asymmetry index that ranges from 0 to 6.

### Tests of Single-locus Effects

Single-locus effects or local effects could lead to spurious HFCs [42], [43], [44]. This alternative mechanism should be carefully evaluated before testing HFCs. Here, we evaluated the potential for single-locus effects in the three steps. First, we assessed the homology of the microsatellite flanking sequences to published Squamata expressed-sequence tags (ESTs). We performed a BLAST search of the flanking regions of each microsatellite locus (sequences available in GenBank; accession numbers are given in Table S2) against the green anole (*Anolis carolinensis*) EST database using the NCBI BLAST suite (http://blast.ncbi.nlm.nih.gov/). We did not find any homologous ESTs between *A. carolinensis* and the flanking sequences of the 10 microsatellite loci. Therefore, the loci we used in this study were not likely to be in functional genes.

Secondly, cryptic population structure is known to produce spurious HFCs [45]. This possibility could be ruled out by testing for heterozygosity differences among populations, or in our case for a single population, to provide evidence that the population is unstructured [46], [47]. The software STRUCTURE 2.3.4 [48] was used to implement Bayesian MCMC inference of a posteriori genetic clusters and detect any cryptic genetic structure in the assumed a priori populations (Mank and Avise 2004). The number of assumed genetic clusters (K) was set from 1 to 10, and 15 runs were performed for each K with 200,000 MCMC iterations (initial 20,000 iterations discarded as burn-in). The results for cluster size K = 1 (LnP(D) = −6621.9) were significantly better than those for K = 2 (LnP(D) = −6910.8, *P* = 0.009) or any higher K values. These results demonstrate that the Jinshan Cape lizard population is unstructured.

Finally, a formal statistical test of single-locus effects was conducted [49]. We performed the F ratio test to determine whether multiple regression incorporating specific effects from each locus (i.e., using heterozygosity at each locus as one predictor, for a total of 10 predictors) explains more variance in a fitness trait than a simple regression using only one predictor, multilocus heterozygosity (MLH). The heterozygosity at each locus was scored as 0 for homozygous and 1 for heterozygous. Missing data were replaced with the mean heterozygosity for that locus [47], [49]. We used the parasite load as the fitness trait in this test because its relationship with lizard fitness (i.e., survival) was formally tested in this study. We log-transformed the parasite load prior to the regression analyses to improve data normality. The results suggest that single-locus multiple regression did not explain more variance in parasite load than did MLH simple regression (*F*_9,129_ = 0.482, *P* = 0.88). Therefore, single-locus effects were not found in this study.

### Tests of HFCs

The two fitness-related traits, parasite load and asymmetry index, were not correlated (Spearman correlation, *P* = 0.62). Hence, their relationships with individual heterozygosity (standardized mean *d* ^2^ and HL) were analyzed separately. The parasite load and asymmetry index data were non-normally distributed. Therefore, the associations between heterozygosity and parasite load or asymmetry index were tested with Spearman correlations. To investigate sex-specific HFCs, we performed Spearman correlations for the whole population, as well as for males and females separately. Because individual heterozygosity increases with standardized mean *d* ^2^ and decreases with HL, the correlations between these two measures and a fitness trait should be in the opposite directions (e.g., a negative correlation between standardized mean *d* ^2^ and parasite load and a positive correlation between HL and parasite load both suggest a positive HFC). Sex differences in parasite load and asymmetry index were examined using Kruskal-Wallis test. All statistical analyses were performed in SAS 9.2.

### Ethics Statement

The lizard handling and processing protocols were approved by the Institutional Animal Care and Use Committee of National Taiwan Normal University (Permit No. 101007). No other specific permits were required because the field studies were not conducted in a protected area, and the sampling did not involve endangered or protected species.

## Results

### Lizard Survival and Parasite Load

**Figure 2:**
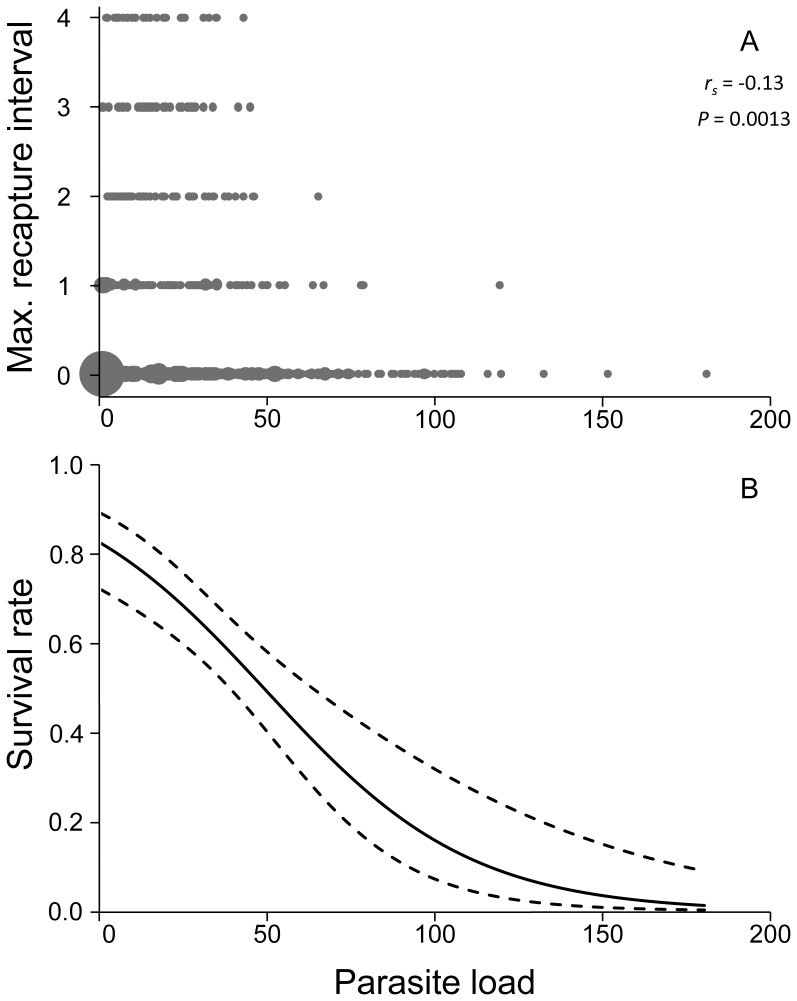
Maximum recapture interval (A) and monthly survival rate (B) of *Takydromus viridipunctatus* against parasite load. This estimation was derived from 1104 adults during the breeding seasons of 2008 and 2009. The maximum recapture interval (in month) decreased with increasing mean parasite load (the mean number of mites of each capture), where the size of shaded circles is in proportion to the sample size (A). The solid and dashed lines (B) denote estimated survival with the 95% confidence intervals, indicating that monthly survival rate decreased with increasing mean number of mites. The covariate plot is based on model 3 in Table 1 and its maximum likelihood estimates, logit(φ) = 1.614±0.318 (SE)−0.032±0.007×(mean number of mites).

Maximum recapture interval (in month) of the 1104 adults represented a negative correlation against parasite load (*r_s_* = −0.13, *P* = 0.0013; Figure 2A), indicating an increase of the mites decreased the probability of the lizard to survive for a long period. Mean number of mites as a covariate for monthly survival of adults in *T. viridipunctatus* was well supported based on the AIC_c_ and LR tests (models 3–6; Table 1). The CJS models with sex-varying slopes of the mean number of mites in lizard survival functions did not significantly improve model fit compared to the models with a constant slope (LR tests: model 3 versus 5, Chi.-sq. = 0.002, *P* = 0.96; model 4 versus 6, Chi.-sq. = 1.913, *P* = 0.17), suggesting that the effects of mites on lizard survival were similar in males and females. In fact, all four models with mean number of mites as a covariate for adult survival (models 3–6; Table 1) estimated negative slopes for mean number of mites (estimated β ± SE: models 3&4, −0.032±0.007; model 5 males, −0.032±0.007; model 5 females, −0.032±0.007; model 6 males, −0.047±0.016; model 6 females, −0.025±0.008), indicating that survival decreased with increasing parasite load for all adult lizards (Figure 2B).

### Individual Heterozygosity and Parasite Load

**Figure 3:**
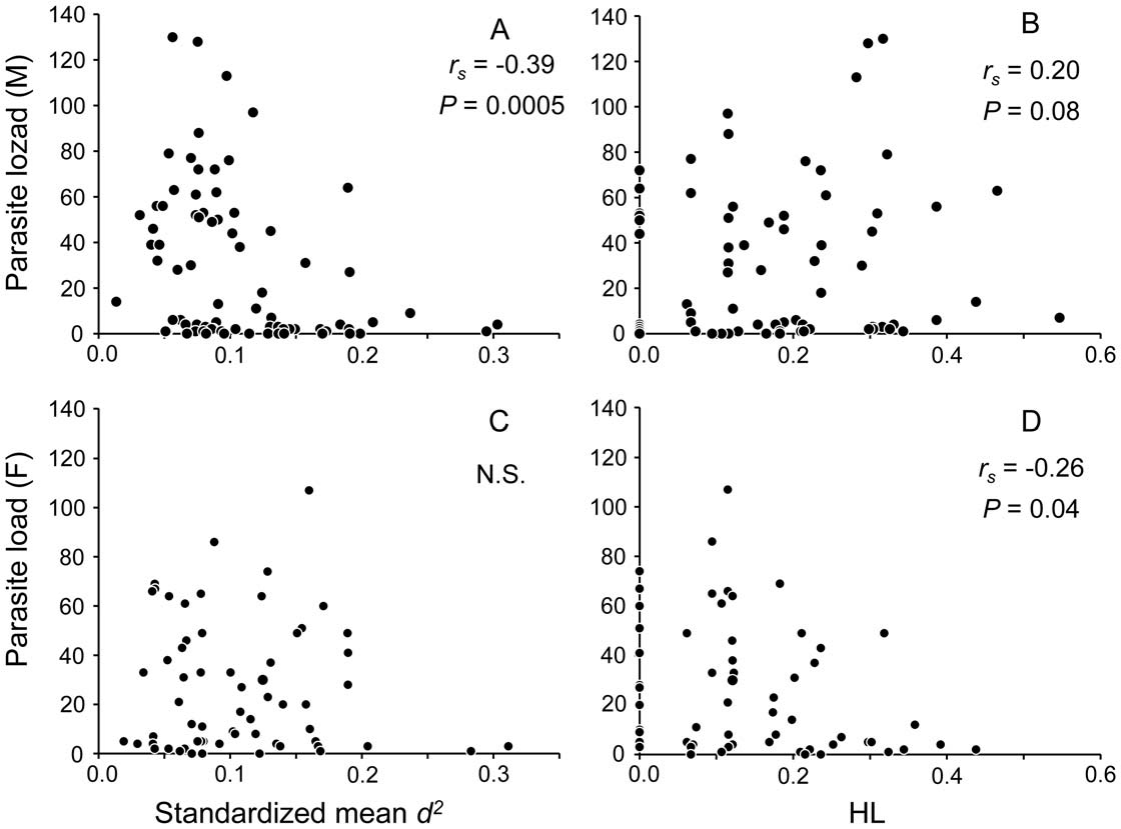
Correlations between individual heterozygosity and parasite load in *Takydromus viridipunctatus*. The parasite load (i.e., the number of the trombiculid mites *Leptotrombidium* sp. on each lizard) increased with decreasing standardized mean *d^2^* (A) and increasing HL (B) in male *T. viridipunctatus*. The parasite load did not change with the standardized mean *d^2^* (C) but decreased with increasing HL (D) in female *T. viridipunctatus*. Each circle denotes one individual.

The 140 lizards collected in July 2008 varied widely in parasite load, ranging from 0 to 130, with a median of 12.5 mites per host. The standardized mean *d* ^2^ and parasite load for the whole population were negatively correlated (*r_s_* = −0.26, *P* = 0.0019, n = 140), and this correlation was driven by the males (males: *r_s_* = −0.37, *P* = 0.0005, n = 78; females: *P* = 0.72, n = 62; Figure 3A and 3C). However, HL and the parasite load of the whole population were not correlated (*P* = 0.97, n = 140), which was a result of the opposite correlation directions between males and females (males: *r_s_* = 0.20, *P* = 0.08, n = 78; females: *r_s_* = −0.26, *P* = 0.04, n = 62; Figure 3B and 3D). When the results from the standardized mean *d* ^2^ and HL were combined, the male lizards showed a consistent pattern in which decreased parasite load was associated with increasing individual heterozygosity (approximately 14% and 4% of the variance in parasite load was explained by the standardized mean *d* ^2^ and HL, respectively). The relationships between individual heterozygosity and parasite load for female lizards, as well as for the whole population, were inconclusive. The male-only correlation between individual heterozygosity and parasite load was not likely to be a result of a higher parasitism risk for males because males and females had similar parasite loads (Kruskal-Wallis test, χ^2^ = 0.88, *P* = 0.35). In addition, both sexes had a similar frequency distribution of parasite load, with a median of 8 (25^th^–75^th^ percentile = 2–52) for males and 18.5 (25^th^–75^th^ percentile = 4–46) for females.

### Individual Heterozygosity and Scalation Asymmetry

**Figure 4:**
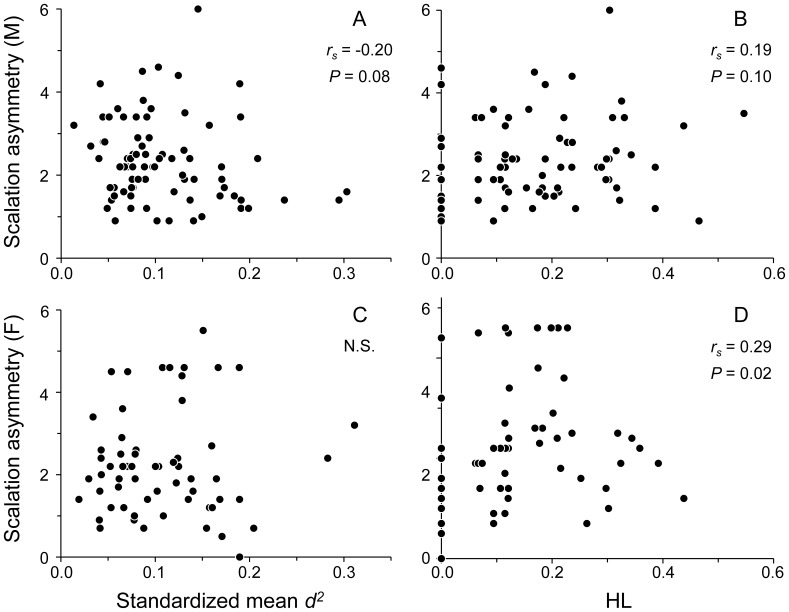
Correlations between individual heterozygosity and scalation asymmetry in *Takydromus viridipunctatus*. The scalation asymmetry increased with decreasing standardized mean *d^2^* (A) and increasing HL (B) in male *T. viridipunctatus*. The scalation asymmetry did not change with standardized mean *d^2^* (C) but increased with increasing HL (D) in female *T. viridipunctatus*. Each circle denotes one individual.

The standardized mean *d* ^2^ and scalation asymmetry index for the whole population were not correlated (*P* = 0.25, n = 140). However, males with increasing standardized mean *d* ^2^ tended to have lower asymmetry (males: *r_s_* = −0.20, *P* = 0.08, n = 78; females: *P* = 0.98, n = 62; Figure 4A and 4C). In contrast, HL and scalation asymmetry index were positively correlated for the whole population, as well as for either males or females alone (whole population: *r_s_* = 0.22, *P* = 0.008, n = 140; males: *r_s_* = 0.19, *P* = 0.10, n = 78; females: *r_s_* = 0.29, *P* = 0.02, n = 62; Figure 4B and 4D). When the results of the standardized mean *d* ^2^ and HL were combined, male lizards showed a consistent relationship between decreased scalation asymmetry and increasing individual heterozygosity (approximately 4% of the variance in scalation asymmetry was explained by the standardized mean *d* ^2^ and HL). The relationships between individual heterozygosity and scalation asymmetry for female lizards, as well as for the whole population, were inconclusive. The male-only correlation between individual heterozygosity and scalation asymmetry was not likely to be a result of higher asymmetry in males because males and females exhibited similar asymmetry index values (Kruskal-Wallis test, χ^2^ = 0.97, *P* = 0.32). In addition, both sexes had a similar asymmetry index frequency distribution, with a median of 2 for both males (25^th^–75^th^ percentile = 2–3) and females (25^th^–75^th^ percentile = 1–3).

## Discussion

This study demonstrated the existence of sex-specific heterozygosity-fitness correlations (HFCs) in *T. viridipunctatus*. In particular, because parasite load had a negative impact on survival (Figure 2), the negative correlation between standardized mean *d^2^* and parasite load and the positive correlation between HL and parasite load in males (Figure 3A and 3B) provide strong support for a male-only, positive HFC. Although we did not observe direct evidence of reduced lizard fitness due to scalation asymmetry, our findings of correlations between standardized mean *d^2^* and scalation asymmetry and between HL and scalation asymmetry (Figure 4A and 4B) also suggest a potentially positive HFC that is specific to male lizards.

Females, on the other hand, did not show a congruent pattern of “heterozygosity- parasite load” correlations, even though they also suffered from reduced survival due to mite parasitism (Figure 2) and even though their parasite loads were not lower than those of the males. It is possible that survival might constitute a larger portion of the male fitness function, whereas reproduction might be more important for the female fitness function. Therefore, the same amount of survival reduction from mite parasitism might have a greater negative impact on male fitness than on female fitness.

Parasites have numerous negative impacts on lizard hosts, such as poorer body condition [50], [51], lower reproductive output [52], slower speed, or smaller home range [53]. However, not all studies found the same fitness reduction effects of parasites in lizard hosts (e.g., [21]). Using capture-mark-recapture methods, we observed one of the few clear demonstrations of a negative impact of mite parasitism on lizard survival and provided empirical evidence supporting heterozygosity advantages for parasite resistance. Although microsatellite heterozygosity is measured at neutral loci, it could reflect heterozygosity at functional loci such as the major histocompatibility complex (MHC) locus [54], [55], which was positively associated with increased disease resistance (e.g., [20], [56]).

The effect sizes found in this study for male lizards (4–14%) were slightly larger than the overall effect size reported in a recent meta-analysis (less than 1%) [6]. It is not surprising for a species with sex-specific HFCs to show weak HFCs at population level. However, this study illustrates that a weak HFC at the population level does not mean that there are no biologically significant heterozygosity advantages in a population. To the best of our knowledge, this is one of the first studies to provide empirical evidence for HFC in lizards (see [7] for a case of a potentially negative HFC in lizards). Our study highlights the importance of investigating sex-specific patterns in HFC studies, particularly for sexually dichromatic populations.

## Supporting Information

Table S1 **Comparison of 16 Cormack-Jolly-Seber models for** ***Takydromus viridipunctatus*** **.**
(DOC)

Table S2 **Locus name, repeat motif, primer sequences, allele sizes (bp), annealing temperature (** ***T*** **_a_), number of alleles, observed heterozygosity (** ***H_o_*** **), expected heterozygosity (** ***H_E_*** **), and statistics of hardy-weinberg equilibrium (HWE) of the 10 microsatellite loci used in this study.**
(DOC)
