# Supplementary material for: Sex-Specific Correlations of Individual Heterozygosity, Parasite Load, and Scalation Asymmetry in a Sexually Dichromatic Lizard
Source: PLoS One. 2013 Feb 25;8(2):e56720. doi: 10.1371/journal.pone.0056720 (PMC3581517; doi:10.1371/journal.pone.0056720)
Supplement: Table S1 — Comparison of 16 Cormack-Jolly-Seber models for Takydromus viridipunctatus . (DOC) [file pone.0056720.s001.doc]

**Table S1.** Comparison of 16 Cormack-Jolly-Seber models for *Takydromus viridipunctatus.*

| Model | AIC_c_ | AIC_c_ weights | Deviance | Number of parameters |
| --- | --- | --- | --- | --- |
| φ_sex_ p_sex×t_ | 1661.3656 | 0.42392 | 50.2279 | 10 |
| φ.  p_sex×t_ | 1661.6403 | 0.36951 | 52.5399 | 9 |
| φ_t_  p_sex×t_ | 1664.7614 | 0.07761 | 51.5823 | 11 |
| φ.  p_t_ | 1666.2398 | 0.03706 | 65.2513 | 5 |
| φ_sex×t_  p_t_ | 1666.6362 | 0.02575 | 53.4572 | 11 |
| φ_sex_ p_t_ | 1666.9676 | 0.01256 | 63.9668 | 6 |
| φ_sex×t_ p_sex×t_ | 1668.4038 | 0.00849 | 49.0782 | 14 |
| φ_t_ p_t_ | 1669.1863 | 0.00561 | 64.1495 | 7 |
| φ_sex×t_ p. | 1670.0164 | 0.00392 | 62.9497 | 8 |
| φ_t_ p_sex_ | 1670.7347 | 0.00315 | 69.7462 | 5 |
| φ_t_ p. | 1671.1724 | 0.00204 | 72.2025 | 4 |
| φ_sex×t_ p_sex_ | 1672.0364 | 0.00000 | 62.9361 | 9 |
| φ. p. | 1688.3806 | 0.00000 | 93.4367 | 2 |
| φ. p_sex_ | 1689.1358 | 0.00000 | 92.1808 | 3 |
| φ_sex_ p. | 1689.5050 | 0.00000 | 92.5501 | 3 |
| φ_sex_ p_sex_ | 1691.1421 | 0.00000 | 92.1722 | 4 |

“φ”: survival; “p”: recapture probability; “sex”: parameter varied by sex; “t”: parameter varied by time; “.”: constant parameter.
