# Supplementary material for: Sex-Specific Correlations of Individual Heterozygosity, Parasite Load, and Scalation Asymmetry in a Sexually Dichromatic Lizard
Source: PLoS One. 2013 Feb 25;8(2):e56720. doi: 10.1371/journal.pone.0056720 (PMC3581517; doi:10.1371/journal.pone.0056720)
Supplement: Table S2 — Locus name, repeat motif, primer sequences, allele sizes (bp), annealing temperature ( T a), number of alleles, observed heterozygosity ( Ho ), expected heterozygosity ( HE ), and statistics of hardy-weinberg equilibrium (HWE) of the 10 microsatellite loci used in this study. (DOC) [file pone.0056720.s002.doc]

**Table S2. Locus name, repeat motif, primer sequences, allele sizes (bp), annealing temperature (*T*_a_), number of alleles, observed heterozygosity (*H_o_*), expected heterozygosity (*H_E_*), and statistics of hardy-weinberg equilibrium (HWE) of the 10 microsatellite loci used in this study.**

| Locus | Repeat motif | Primer sequences(5’-3’) | Allele sizes (bp) | *T*_a_(℃) | No. of alleles | *H_O_* | *H_E_* | *P* (HWE) | GenBank Accession no. |
| --- | --- | --- | --- | --- | --- | --- | --- | --- | --- |
| TF013 | GATA | F: ATTCCGTGGCACTTGGCAGG | 178-230 | 55 | 13 | 0.830 | 0.841 | 0.15 | DQ012386 |
|  |  | R: TGGCTCATAGTATTGGCTTGC |  |  |  |  |  |  |  |
| TF046 | GATA | F: ACAGAAGAACTTGGGTCTCCAG | 211-263 | 61 | 33 | 0.943 | 0.946 | 0.34 | DQ012388 |
|  |  | R: GCTCTCCAGCCAGGTGTCAC |  |  |  |  |  |  |  |
| TF064 | GAAA | F: CGACTCATCTCCCGTGCCAG | 230-290 | 55 | 17 | 0.871 | 0.887 | 0.48 | DQ012390 |
|  |  | R: ATTTCTCAAGGTGACATTGG |  |  |  |  |  |  |  |
| TF098 | GAAA | F: ATCTAAATGGTCCACTGATC | 209-265 | 55 | 14 | 0.833 | 0.888 | 0.23 | DQ012394 |
|  |  | R: ACTGAAGGGATGGCAATGAG |  |  |  |  |  |  |  |
| TF100 | GAAA | F: GTTCCCTGGGGCTACCACAG | 202-298 | 55 | 20 | 0.856 | 0.899 | 0.02 | DQ012395 |
|  |  | R: AATGGATAACTGGCTTCCTG |  |  |  |  |  |  |  |
| TF102 | GAAA | F: CAGCCTGGTATCACTACTGCAC | 204-252 | 55 | 13 | 0.894 | 0.933 | 0.55 | DQ012396 |
|  |  | R: TCCAGAGCAATTGAAATGCCTG |  |  |  |  |  |  |  |
| TF130 | GAAA | F: AAGAACTGTCkGGCTCTGTC | 166-262 | 55 | 12 | 0.886 | 0.888 | 0.79 | DQ012398 |
|  |  | R: TGTCTGAAATCAGTGGCAAC |  |  |  |  |  |  |  |
| TF159 | GAAA | F: CCTGTTAGATTCTGCCATTC | 224-324 | 52 | 22 | 0.906 | 0.905 | 0.52 | DQ012403 |
|  |  | R: TGCCATACAAATTCCCACACC |  |  |  |  |  |  |  |
| TF163 | GAAA | F: TGGAAACACTGGCAAAGGAG | 222-282 | 55 | 19 | 0.814 | 0.910 | 0.01 | DQ012404 |
|  |  | R: TTCTCTCATTCGGGGGTATC |  |  |  |  |  |  |  |
| TF167 | GAAA | F: GTGACCTCGTCCATGTGTCG | 261-323 | 55 | 15 | 0.764 | 0.737 | 0.80 | DQ012405 |
|  |  | R: CAAAGTCTATCGCAGCACTG |  |  |  |  |  |  |  |
